# Deterrence Rediscovered: NATO and Russia

**DOI:** 10.1007/978-94-6265-419-8_3

**Published:** 2014-12-15

**Authors:** Sten Rynning

**Affiliations:** 3 Faculty of Military Sciences, Netherlands Defence Academy, Breda, The Netherlands; 4 Faculty of Military Sciences, Netherlands Defence Academy, Breda, The Netherlands; University of Southern Denmark, Odense, Denmark

**Keywords:** North Atlantic Treaty Organization (NATO), collective defence, grand strategy, reassurance, deterrence, access denial, horizontal escalation, burden sharing

## Abstract

The North Atlantic Treaty Organization (NATO) is back in the business of deterring aggression on the part of Russia. This return to great power deterrence has brought widely acknowledged military challenges related to power projection, force modernization, and burden sharing but also and notably a political challenge of defining NATO’s collective political ambitions for a continental order in which Russia will not become like the West. Like during the Cold War, the most convincing posture for NATO has become one of deterrence by punishment, building on a fairly dynamic military ability to strike Russia at a point of choosing, as opposed to defending every entry point to Alliance territory. However, NATO, not sure of what political order it represents, struggles to read Russia’s political character and intent and size its military posture accordingly. NATO’s political deficit effectively robs it of a middle ground from where it can build its military posture and invest in its upkeep. In the 1960s, NATO forged such a middle ground as an essential platform for strategic adaptation; today, NATO’s full deterrence posture is suffering from the absence of such a middle ground. Thus, a comprehensive politico-military posture of deterrence vis-à-vis Russia will require NATO’s reengagement with its own political fundamentals.

## Introduction

“We have debated this endlessly, and it is just not easy,” one North Atlantic Treaty Organization (NATO) official remarked in early 2015 when asked to identify the principles underpinning NATO’s new deterrence posture.^1^ Russia had in the course of 2014 “fundamentally challenged our [NATO’s] vision of a Europe whole, free, and at peace”, and NATO spoke boldly of its determination to remain the “essential source of stability in an unpredictable world”.^2^ However, whether its deterrence would be by punishment or denial, how it would build on US extended deterrence, and how it would tie into dissuasion and persuasion, that was the question.

NATO authorities could take solace in the fact that, throughout Alliance history, the establishment of deterrence had been a delicate affair. The very first Strategic Concept for the Defence of the North Atlantic Area, of November 1949, put the creation of a “powerful deterrent” at its heart.^3^ Still, the military chiefs of the Alliance responsible for strategic guidance and regional defence plans had to cope with changing political and organizational conditions within the Alliance—Greece and Turkey acceded to the treaty; Western Germany was on the horizon as a defence obligation; and NATO gained major commands to take over from its disparate regional planning groups—and then the fact, as they dryly noted, that the Strategic Concept “contains no assessment of the capabilities or possible intentions of the enemy”.^4^

Through generations of debate illuminated earlier in this volume,^5^ the scholarly deterrence community has been brought back to this square one where NATO chiefs at one time found themselves and where deterrence first and foremost is a question of tailoring threats to specific actors and their political desires. Fittingly, the argument of this chapter is that NATO, today as during the Cold War, is largely wedded to deterrence by punishment and maintains a solid and fairly dynamic military posture. However, then as now, NATO’s political ability to read and adapt to Russia’s political character and intent is limited.

The chapter builds on the distinction between strategic planning and strategic improvisation, arguing that NATO’s contains a far greater degree of improvisation than its reliance on plans, policies, and procedures indicate.^6^ The ability to improvise in the face of an agile adversary is a quality, but, as we shall see, NATO improvisation occurs not least because of shortcomings in the Alliance’s ability to set its own political compass. In terms of the three distinct components of grand strategy—“grand principles”, “grand behaviour”, and “grand plans”^7^—NATO’s strong suit is unquestionably the pattern of military deployments and exercises that underpin “grand behaviour”, whereas its distinct weakness is its inability to settle on “grand principles” and apply them in a reading of Russia’s ambitions and intent. NATO’s “grand plans” are thus sandwiched between solid military practice and the improvisation that flows from limited political abilities.

The literature on NATO deterrence of Russia post-2014 quite rightly highlights NATO challenges in terms of limited muscle and institutional memory when it comes to joint high-intensity warfare;^8^ a political geography that favours Russian interior lines and confounds NATO plans of reinforcement;^9^ and discomfort with a new interface between conventional and nuclear deterrence.^10^ As the discussion of NATO’s “grand behaviour” and “grand plans” will outline, NATO, rather than plugging every hole in its armour, must develop a posture of strength that unmistakably promises punishment in relation to Russian aggression. This is not simple, and among the issues to navigate are burden sharing and a common approach to manoeuvre warfare and new technology,^11^ and ultimately, and as this chapter argues, it presupposes a clear understanding of the adversary. This is where NATO’s forced improvisation should be of concern because it tells the story of Alliance hesitation on the key prioritization of politico-strategic intransigence^12^ versus dialogue.^13^ Whether one or the other should have priority in a grand strategic effort to bolster deterrence can only be determined after careful deliberation on the nature of the threat, and in this regard, NATO comes up short.

Sections 3.1 and 3.2 below examine these political shortcomings. Section 3.3 examines NATO’s “grand behaviour”, while Sect. 3.4 turns to NATO’s politico-military planning—its “grand plan”. The conclusion considers implications for NATO strategy.

## Know Thyself, NATO

NATO is experiencing a gap between liberal values to which it is committed by treaty and a resurgence of national values that are not necessarily liberal. What NATO stands for is therefore up for debate, and the ramifications hereof run through all dimensions of its collective deterrence posture. In its official declarations, NATO remains steadfastly committed to “democracy, individual liberty, human rights, and the rule of law”—as reflected in its 1949 treaty and its London Declaration of December 2019 celebrating the Alliance’s 70th anniversary.^14^ The irony, though, is that NATO heads of state and government gathered in London a full eight months following this anniversary, and then in a lean and quick format that did not warrant the label “summit” but merely “meeting”, because of underlying tensions between national values and outlooks.^15^ At NATO’s highest political level, discomfort about what NATO stood for had become patently visible.

NATO was thus in a situation where its conceptual coordinates were unable to guide allies in their search for grand objectives for grand strategy. Two such grand objectives were possible. One was to seek an accommodation with Russia along current lines of political influence in order to facilitate an explicit balance of power at the heart of Europe’s security order. It would not imply the rollback of NATO, though some realists might advocate this course of action as a consequence of their distinctive criticism of NATO enlargement,^16^ but a halt to the liberal ambition to aid in the transformation of Russian society and government and to ultimately build a continental order of liberal democracy.^17^ The other option would be the inverse hereof—to support the aspirations of people wherever they may be for self-determination and greater freedom, and to use NATO as a mechanism for extending liberal-democratic norms into the former Eastern bloc.^18^ It would be tantamount to harnessing power for political aspiration, where the other option would be to restrain aspiration for balanced power.

Multiple implications flow from these grand objectives. A strong aspirational commitment on the part of NATO would maintain enlargement in process, cause political discomfort in Moscow where it would be seen as invasive, and lead Moscow to instrumentalise conflicts outside of the Euro-Atlantic area for the purpose of diluting NATO. It would raise the requirements for NATO deterrence and, because the United States remains the sine qua non of collective defence and deterrence in NATO, markedly restrain the scope for the development of a European pillar.

NATO has been zigzagging on these objectives and remains perfectly willing to kick the can down the road, notably in regard to the membership prospects of Ukraine and Georgia. “We agreed today that these countries will become members of NATO,” is how NATO heads of state and government put it in 2008,^19^ but they have since postponed further formal steps with reference to political circumstance and conditions attached to the Alliance’s Membership Action Plan. Ukraine and Georgia were thus not mentioned in the London Declaration of December 2019, and on Russia NATO remains steadfastly committed to the partnership framework, a Founding Act, agreed to in 1997.^20^ How NATO can commit to the NATO-Russia Founding Act goal of an “undivided Europe” and simultaneously offer Ukraine NATO membership, strongly opposed by Russia, is the bullet NATO is dodging.

There is a direct link from NATO’s inability to emphasize one or the other grand objective to the ongoing wider debate within NATO on whether collective and national interests can stand in opposition to one another. President Trump’s “America First” agenda along with his reluctance to embrace NATO’s Article V collective defence commitment and his contrasting frequent harsh criticism of allies’ contributions lies at the heart of this agenda.^21^ At issue is the extent to which the allies themselves can anchor their national interests in a common liberal framework, as opposed to having exclusive national interests that only on occasion coincide. In Henry Nau’s perceptive assessment, the risk is one of nationalism within the NATO area developing into antagonisms based on blood (ethnicity), history (culture), soil (territory), or creed (ideology), which leads Nau to suggest pathways for “conservative internationalism” whereby traditional liberal concerns with free government and society get funnelled through renewed and reinvigorated national interests.^22^ Conservative internationalism suggests that NATO can build on both liberal and national values: however, it would imply a diminished reliance on NATO as an institution, which to nationalists has become akin to an iron cage of rules and ties that inhibit political thinking and provide cover for freeriding, and an enhanced role for strong nations that step out front in the “political alliance” rather than the “constraining organization” (the “O” in NATO).

Such an alignment with a conservative merger of liberalism and nationalism would imply at least NATO’s partial alignment with the balance-of-power option: the NATO institution would no longer serve as the anchor of liberal-democratic norm export and NATO nations looking to navigate a wider global context of Chinese power and other emerging issues would be inclined to want to reduce systemic tension with Russia. However, the attractiveness of such a set of conceptual coordinates is complicated by Russia’s 2014 annexation of Crimea. The Alliance is strong in its opposition to this land grab and to any suggestion that there can be a return to “business as usual” for as long as Russia maintains it. The prospect of caving in to Russian coercion and manipulation is distinctively unappealing across the Alliance. Unity on these points feeds a military deterrence posture we shall encounter below. However, it also feeds great uncertainty on the political objectives that deterrence is supposed to serve, and which, for now, remain the liberal set of values written into the 1997 Founding Act. Maintaining the 1997 vision of an undivided Europe is a way to stonewall Russian manipulation, of course, and the Alliance willingly exploits this irritant to Russian diplomacy, but it has de facto also become a substitute for moving NATO consensus on East–West political objectives forward.^23^ President Trump’s imbroglio in Russia investigations and an impeachment procedure motivated by his actions in Ukraine merely underscores how politically difficult it is for the Alliance as a whole to move forward politically on these issues.

Hal Brands has likened grand strategy to “the intellectual architecture that lends structure to foreign policy”.^24^ Going by this definition, NATO’s grand strategy, within which Russia deterrence is embedded, is fractured in its intellectual architecture. NATO allies are unsure of their own value-base: of how longstanding liberal principles and renewed nationalism can coexist and perhaps even reinforce each other within the Alliance. This uncertainty inhibits collective reflection, and thus policy, on intransigent issues related to Russia. The default intellectual architecture NATO leans on dates back to 1997, and while this is politically convenient in terms of stonewalling Russia and buying time for NATO’s internal diplomacy, it does little to give political direction to the Alliance’s renewed deterrence posture.

## Know Thy Enemy

As a consequence of NATO’s uncertain value base, the Alliance struggles to come to grips with Russia’s political intent. There can be no question that the allies are united in their opposition to Russia’s annexation of Crimea. Equally, there is no question that the Alliance perceives and reacts to Russia’s “new generation warfare” that is essentially a strategy of coercion centred on the “informational space” of Western societies.^25^ New generation warfare is a cross-domain tool for compelling and deterring Western policy, and it makes no distinction between war and peace—a building block for Western thinking.

NATO has confronted Russia’s new thinking in a number of policy respects, including societal resilience, enhanced intelligence cooperation, cyber security, and rapid decision-making. NATO’s challenge lies elsewhere, namely in respect to the holistic assessment of Russia’s political nature and intent on which policy must be based. A series of background interviews with NATO officials (conducted in May and December 2019, as well as January 2020) convey the imagine of an Alliance that at the decision-making level—in the North Atlantic Council (NAC)—tends to be circumscribed and reactive. The NAC certainly addresses Russia but by and large in specific contexts, be it in regard to hypersonic weapons, intermediate range missiles, Black Sea presence, or other pressing issues. The advantage hereof is that NATO is able to interact with some agility with Russia without getting bogged down in difficult discussions of political philosophy. NATO has always heralded its operational, as opposed to philosophical, character, setting it apart from, say, the European Union. However, the disadvantage is that NATO, never really confronting the sum total of Russian actions, can come to rely on crude assessments of or mere assumptions about the nature and architecture of Russian ambitions.

NATO does possess institutionalized mechanisms designed to deliver holistic assessments of Russia—it is just that they connect poorly to the decision-making level.^26^ The Joint Intelligence and Security Division established in 2014 is one such mechanism. The division at NATO’s political-military headquarters does not gather its own intelligence but coordinates that offered by nations and integrates it into collective overviews of Russia’s policy and actions. Such coordination is especially relevant in regard to “hybrid” threats that sow seeds of confusion in allied informational space. In addition, the deputy secretary general is in a unique position to guide the occasional and very scripted encounters between Russia and NATO in their NATO-Russia Council. Rose Gottemoeller, a Russian-speaking American diplomat, came to this post in 2016 and stayed on for three years, and by virtue of her extensive insight into Russian politics and security policy set a high standard for the position that her successors must seek to imitate. The Deputies’ Committee—composed of the deputies to NATO ambassadors and in many ways the workhorse of the headquarters—holds monthly informal talks under the heading of “understanding Russia”, and sometimes they invite external experts to share insights. Finally, and importantly, these collective mechanisms are open to the substantial Russia-knowledge that especially the larger NATO nations possess—and in particular the Quad (the United States, Britain, France, Germany)—which brings us back to the political level and the disconnect between collective expertise and political deadlock.

There was always a tension between NATO consultations on the one hand, which by nature are collective and cumbersome, and even more so in an enlarged Alliance, and informal big power consultations on the other. The Quad is a case in point, having emerged to manage German issues at the point where occupation rule came to a conclusion, in 1955, and then surviving and even prospering as a go-to format for big power coordination. The Quad became a “portable” format that the Quad countries deliberately kept apart from NATO in order to preserve confidentiality and flexibility.^27^ This fault line between collective institutions and big power insight and diplomacy persists, but today it is aggravated by the underlying and widespread tension over basic NATO values. In other words, the Quad is as inflicted as other institutions by political disunion and is unable to come to the rescue of NATO.

NATO has appealed to the tried and tested dual-track approach of emphasizing both defence and dialogue, dating back to its Harmel doctrine of the late 1960s—after Belgian foreign minister Pierre Harmel—that offered the Soviet Union political dialogue within a framework of solid allied defence. Today, NATO sometimes adds “deterrence” to the equation and therefore speaks of 3 Ds—deterrence, defence, and dialogue.^28^ However, in recognition of the poverty of dialogue without internal agreement, NATO in December 2019 agreed to undertake a “forward-looking reflection process” to “further strengthen NATO’s political dimension including consultation”.^29^

The mandate and composition of the group that must undertake this reflection process is contentious, though. The idea of setting up such a group was German, introduced into Alliance diplomacy in November 2019 to defuse tensions flowing from French President Macron’s statement that NATO was “brain dead”, but little was agreed apart from the lead role of their secretary general, Jan Stoltenberg. In the wake of the London meeting the idea was floated to turn this process into a precursor for revising NATO’s capstone Strategic Concept, which would have forced NATO to define its broader Russia view, among other things, but this broad and ambitious idea was quickly and effectively killed.^30^ What the reflection process will deliver remains to be seen, but it will likely be a workmanlike anticipation of how NATO can adjust to the outcome of the US presidential elections in November 2020. While NATO’s broad understanding of the challenge posed by Russia is solidly anchored, NATO’s holistic and detailed assessment of Russia’s political nature and intent is lacking. There is ample expertise on Russia inside NATO and particularly within certain allied capitals, but the political framework for mobilizing and integrating it into an allied strategic assessment is weak and therefore ineffectual.

## Grand Behaviour

In early 2020, the US Army began the exercise Defender-Europe 20, which involved the deployment of a division-size combat-credible force from the United States to Europe. Up to 30,000 US and allied troops would be involved in this “from fort to port” exercise that from a land forces standpoint, Lt. Gen. Cavioli, commander of US Army Europe, argued, shows how “the demonstration of our collective defence is our best deterrent”.^31^ With this, NATO was flexing its US-based follow-on force muscle that define the core of its ability to conduct major joint operations in Europe. Short of nuclear war, this capacity captures the essence of a NATO deterrence by punishment posture.

NATO had built up this capacity for deterrence by punishment with considerable care since 2014. Enhanced US investment in extended deterrence has formed the backbone hereof. This effort began in 2014 with the so-called European Reassurance Initiative which in the course of 2017 was upgraded to a European Deterrence Initiative, which has allowed the funding of a heel-to-toe presence (i.e., continued but rotational and not permanently stationed troops) of an Armoured Brigade Combat Team with enablers, a Combat Aviation Brigade, an Army Battalion, and a range of supporting infrastructure and exercise investments. What began as a one-year $1 billion emergency response to Russian aggression in 2014 had by 2020 grown into an ongoing $6 billion deterrence program and a primary funding source for the US European command.^32^

NATO allies have complemented this US investment in a number of ways. First of all, they have put more defence money on the table: NATO Europe and Canada invested $313 billion in defence in 2018 compared to $272 billion in 2014. Moreover, in June 2018 they committed to a NATO Readiness Initiative according to which they would have 30 battalions, 30 air squadrons, and 30 naval combat vessels ready to use within 30 days—with the details hereof being worked out through 2020. Also, in 2018, the allies agreed to reform their command structure, re-introducing the North Atlantic command in Norfolk, Virginia, and introducing a new support and logistics command in Ulm, Germany, both designed to secure lines of communications and enable transatlantic reinforcements to NATO’s eastern frontiers. In addition, the reformed command structure gained a Cyberspace Operations Centre, following NATO’s 2016 decision to recognize cyberspace as a domain of operations.

All these measures bolster NATO’s conventional deterrence by punishment posture. The ultimate source of deterrence by punishment is nuclear, and NATO has in this regard undertaken significant but still limited steps. The Alliance revived its nuclear consultations in the course of 2015, including a nuclear consultation exercise based on an Article 5 (collective defence) scenario, and its Warsaw Summit communiqué contained an unprecedented number of references to nuclear forces, even if they mainly rehashed past language of restraint (i.e., “The circumstances in which NATO might have to use nuclear weapons are extremely remote”).^33^ NATO language will have to change and reflect doctrinal adaptation, according to the warning of two prominent analysts: Russian doctrine is premised on the early introduction of nuclear weapons in armed conflict, and NATO must do away with its “extremely remote” doctrine in favour of a “decisive response” doctrine; and this doctrine must underpin NATO’s ability to strike into Russia with conventional weapons to deter a limited Russian “land grab” operation in Estonia or elsewhere, they contend.^34^ In short, NATO has taken steps to reinforce its nuclear deterrence but still has some work cut out at these upper levels of the ladder of escalation.

Deterrence by denial (i.e., an ability to deny Russian objectives by defensive measures) is only possible for NATO at the lower rungs of this ladder, and NATO has not been idle here either. In fact, most of the early measures taken by NATO in response to the annexation of Crimea fall into the deterrence of denial category and centre on rapid reaction capacities, especially in the shape of a NATO Response Force (NRF) upgraded for deterrence purposes. The NRF now has a reinforced, quicker spearhead—a Very High Readiness Joint Task Force potentially up to 13,000 troops strong, and then two complementing brigades with support (each 13,000 strong) forming a layered, sizeable reaction force explicitly linked to collective defence purposes and regularly exercised in Eastern Europe and the Baltic states.^35^ In 2016, in response to the foreseeable difficulties of projecting mainly Western forces into zones of conflicts close to Russia, NATO decided to established an “enhanced forward presence”—four multinational battalion-sized battle groups—in the Baltic states and Poland, and a “tailored forward presence”—mainly naval forces—in the Black Sea region.

Whether these forces can credibly “deny” Russian objectives in the case of limited war is a bone of contention. Most observers and sometimes NATO itself employ the descriptor “tripwire” to these forces, thus indicating that they are triggers that promise to unleash NATO’s big guns and therefore part and parcel of deterrence by punishment. However, US diplomats (interviewed on background) feel more confident that the US battalion embedded (in Poland) in the collective forward presence posture would actually be able to fight and survive, and thus deny Russian objectives. That may be so, in which case the conclusion is that NATO has a moderate-to-low—and geographically focused—capacity for deterrence by denial and then a more general and impressive capacity for deterrence by punishment.

NATO’s unquestionable capacity for deterrence by denial is rather found at the level of grey zone, non-kinetic conflict. In this regard, NATO has upgraded not only its cyber defences and enhanced intelligence coordination, as mentioned, but has enhanced coordination with the European Union on hybrid threats, with a 2016 joint declaration leading to a common work program and a collaborative Centre of Excellence for Countering Hybrid Threats, located in Helsinki, the 2016 adoption of societal resilience benchmarks that, while mostly falling outside NATO’s political-military remit, nations must meet, and finally the decision in 2018 to organize counter-hybrid support teams that can tailor assistance to individual allies and circumstances.^36^

NATO’s full range of actions in response to Russia’s 2014 annexation of Crimea—a range to which this brief overview can do only limited justice—thus combines deterrence by denial (grey zone conflict, societal resilience, reaction and forward deployed forces to counter limited land grabs) and deterrence by punishment (the full chain of reaction and deployable forces, from conventional to nuclear). NATO’s strong suit is the military piece of this posture, but it has considerably adapted to grey zone conflict scenarios in an effort to achieve a comprehensive deterrence posture vis-à-vis Russia’s unified (kinetic and non-kinetic) and uninterrupted (all domains, in war and peace) doctrine of “new generation warfare”.^37^

## Grand Plans?

NATO’s robust military response to Russian aggression is ultimately dependent on coherent politico-military guidance. In this regard NATO benefits from the routine and leadership embedded in its integrated military command structure, capped off by the double-hatted US general serving as both NATO’s supreme allied commander (SACEUR) and commander of US forces Europe (EUCOM), currently General Tod D. Wolters. A number of challenges related to political priorities beset this planning, however.

NATO has adopted a revised “military strategy” (MC 400/4), which is a first since its adoption of its flexible response strategy in 1967 (MC 400/3). The long interlude can be explained by the appropriateness of MC400/3 through the remaining Cold War years and then NATO’s post-Cold War need to improvise “other than war” crisis response operations, which had limited import for the strategy’s peer-to-peer focus. This changed in 2014 with Russia’s aggression in Ukraine, and by 2017 NATO’s Military Committee, composed of allied Chiefs of Defence, who was tasked to work on an integrated new military strategy fit for purpose. The Military Committee was able to approve this new strategy, MC400/4, in May 2019.^38^ Next steps are to operationalize it, secure political approval by Ministers of Defence in June 2020, and enable SACEUR to draw up concrete plans and directives for his subcommands.

Two key concepts inform the new military strategy: theatre-wide approach and horizontal escalation, with both largely tying into NATO’s overarching strategy of deterrence by punishment. The theatre-wide approach is NATO’s military answer to the complexity of NATO geography: it is to say that NATO will not divide its planning and forces regionally but instead insists on having an integrated and seamless approach to defence and deterrence in the Euro-Atlantic area. NATO initially responded to Russia by drawing up Graduated Response Plans (GRP) that were geographically compartmentalized, covering segments of NATO’s frontier from the North Atlantic through Central Europe to the eastern Mediterranean. A key weakness in this response was NATO’s limited ability to think and move across these GRP compartments, leading to the search for a truly “theatre-wide” capacity for defence and deterrence. The answer lay in the other key concept, horizontal escalation, by which NATO means military forces able to move across NATO territory at the “speed of relevance”.^39^ The aforementioned 4 × 30 Readiness Initiative along with the revised command structure are key enablers hereof.

NATO’s military strategy largely builds on deterrence by punishment because of its theatre-wide and therefore asymmetrical threat of escalation. It leaves Russia guessing where NATO could respond to an attack, simply promising Russia that NATO has this asymmetrical capacity and intent. There remains an element of deterrence by denial in so far as NATO maintains high readiness forces along with enhanced forward presence forces to bolster its territorial defence, particularly in the Baltic area; for larger threats of Russian force, though, NATO resorts to its counter-threat of theatre-wide escalation and therefore deterrence by punishment.

It is with a degree of timidity that NATO agreed to this posture. Initially, in 2014–2015 when NATO agreed to the GRPs, the allies were divided between two opposite desires, one of developing Cold War-style elaborate defence plans against Russia, and another of sticking to broad stroke contingency plans. The GRPs were a compromise: they had distinct geographies and reaction forces attached to them, but the reaction forces were limited in number (essentially, the NATO Response Force), the GRPs were not coordinated, and there were only contingency plans for follow-on forces. SACEUR has been a primary institutional actor in the effort to solidify this compromise and move NATO to a more stringent—more deterring—posture of theatre-wide punishment. In December 2018 SACEUR delivered his “strategic thoughts” on the draft military strategy that had come out of internal consultations between NATO military authorities.^40^ SACEUR used the occasion to great effect, challenging the allies to place the credible deterrence of Russia at the heart of their thinking. Put differently, the tendency to think broadly and inclusively, to give as much thought to counterterrorism and stability operations as to Russia, and to settle for diplomatically appealing but ineffective GRPs, muddled NATO’s posture and failed to offer a robust allied response to Russia’s challenge.^41^

While it was always impossible for political reasons in a diverse alliance to give sole attention to just one threat, namely Russia, SACEUR’s intervention did succeed in upgrading allied thinking on this particular threat. The military strategy that the Chiefs of Defence approved in May 2019 thus involves a range of threats—from Russia over counterterrorism to stability operations—but its centre of gravity is the emphasis on theatre-wide defence and horizontal escalation that SACEUR had in mind.

The military strategy ultimately depends on political support for its success, of course. Here the encouraging news is that NATO allies have approved not only the military strategy but also some of the key measures that enable it: from the readiness initiative over the enablement focus within the command structure to an intensified training and exercise schedule. As might be expected, though, the allies remain preoccupied by burden sharing issues that to a degree could delay implementation. The 4 × 30 readiness initiative is particularly cumbersome to stand up: readiness is costly because forces are paid to be on standby, just as training and exercises are costly; it is not clear how these ready units will combine and be integrated in the command structure, meaning SACEUR’s command authority remains undefined as do implications of the ready forces in NATO’s Response Force; and, finally, it is not clear how big a role US forces will play in the 4 × 30 package. The US approach is to steer clear of these wider questions—that some European allies are wanting to address right away—in order to keep the focus stringently on the readiness initiative itself. Put differently, the United States does not want some allies to be able to hide behind an organizational screen and defray costly reforms at home.

The political commitment to following through with the military strategy is therefore the question. NATO has politically chosen to emphasize deterrence by punishment: this is its theatre-wide approach with flexible, exercised, and ready forces. The alternative would be to deter by denial by identifying critical strong points that Russia would need to attack and building up strong defences around them. The alternative is appealing because it relies less on the ultimate deterrent of nuclear weapons.^42^ However, given NATO’s geography—where Russia has formidable access denial capacities in some areas and can ignore geographical constraints in others, considering its broad “new generation warfare” toolbox, and where every NATO ally sees itself as a valuable strong point—NATO cannot politically opt for denial. NATO has almost by default, though with some timidity, as we saw, opted for deterrence by punishment. Its military command has produced a coherent plan—a military strategy—for realizing this posture, but as with any military strategy, it is hostage to the clarity and collective strength offered by the Alliance’s conceptual coordinates with which this chapter began.

## Conclusion

NATO has committed largely to deterrence of Russia by punishment. The Alliance maintains certain elements of deterrence by denial—notably resilient societies and a degree of strong point defence (i.e., enhanced forward presence), but with the understanding that Russia cannot be denied if it throws its full military weight into an attack on allied territory at a point and time of its choosing. NATO’s commitment to theatre-wide asymmetrical and horizontal escalation, premised on trained and tested response and reaction forces and an enablement command, follows. NATO’s is a strategy of punishment intended to leave Russia in the dark as to the timing and nature of NATO’s response to its aggression.

NATO has arrived at this posture gradually, moving from measures to reassure exposed allies to a posture of deterrence of Russia. In this movement, NATO has varied its emphasis on immediate reaction forces, in-place forces, and, now, reaction forces for the European theatre, just as it has varied its stance on contingency and defence planning. NATO has slowly but surely engaged a debate of its nuclear posture and doctrine, dusting off nuclear consultation mechanisms and exercises, though critics will say that there is room for improvement here, an argument that could applied equally to the conventional and nuclear domains. Still, in terms of “grand behaviour” and “grand plan”, NATO’s design for and commitment to deterrence by punishment is clear and emerging.

The conceptual coordinates flowing from “grand principles” are trickier. NATO allies are in disagreement on the extent to which it is possible to coexist with Putin’s Russia in a balance of power arrangement or, inversely, the extent to which continental order depends on the transformation of the character of Russia’s political regime. Worryingly, uncertainty in regard to Russia is tied to, and in many ways flows from, uncertainty within the Alliance on NATO or Western values. Nationalist doctrines are challenging classical liberal doctrines in many allied capitals, and what this means for NATO strategy is simply unclear. NATO’s collective response has been to stick to old guns—the partnership vision of 1997—and attend to the military “behaviour” and “plans” on which allies can agree.

In the Cold War, NATO’s flexible response strategy (MC 400/3) reflected a political compromise. The United States preferred as much conventional defence as possible, paid for by European allies, and thus as much deterrence by denial as possible. The European allies preferred deterrence by punishment and thus NATO’s threat of quick and flexible escalation to nuclear war, effectively tying the fate of Western Europe to that of the United States. MC 400/3 captured the middle ground. Today, European allies are equally committed to deterrence by punishment but in doubt on how much to deliver, partly because the widespread refusal to fall back on flexible nuclear deterrence raises the costs of conventional reform, partly because they are not in agreement on the nature of the Russia threat. The United States is pushing the European allies to undertake these reforms and investing its own conventional muscle in Euro-Atlantic deterrence, but it is so mired in domestic disagreement on Russia and foreign policy that it can offer NATO little politico-strategic guidance here. There is thus no political middle ground from where MC 400/4, NATO’s new military strategy, can be built.

NATO continues to face critical decisions in terms of future military technology, defence plans, and training and early warning regimes, and the challenge hereof should not be diminished by the encouraging reading offered here that in terms of “strategic plans” and “strategic behaviour”, NATO has managed to put together a fairly credible and dynamic deterrence posture. Less encouraging and ultimately more alarming is the conclusion that NATO’s ability to dedicate its political mind to reading the character and intent of its rival is limited. Worryingly, the political and economic fallout from the 2020 Corona pandemic could likely exacerbate the internal political fractures that explain this poor condition.^43^ NATO’s political condition is thus of direct and immediate consequence for its deterrence posture, and the building of a political middle ground for its military strategy should be a primary concern for Alliance leaders.

## Footnotes

1 Background interview by author at NATO headquarters.

2 NATO 2014, para 1.

3 Donnelly 1949.

4 Standing Group and Military Representatives Committee 1952.

5 Mazarr 2018.

6 Popescu 2017, 2018.

7 Silove 2018.

8 Kroenig 2015; Sweijs and Osinga 2019.

9 Shlapak and Johnson 2016; Veebel 2018; Zapfe 2017; and Zapfe and Haas 2016.

10 Durkalec and Kroenig 2016; Larsen 2019.

11 Simón 2016; Sweijs and Osinga 2019.

12 Freudenstein 2016; German 2017.

13 Kühn 2015.

14 NATO 2019.

15 Burns 2019; Cook 2019.

16 Mearsheimer 2014.

17 Kissinger 2016; Rynning 2015.

18 Gheciu 2005; Thies 2009.

19 NATO 2008.

20 NATO 1997.

21 Kaufman 2017; Schreer 2019.

22 Nau 2013, 2018.

23 Ringsmose and Rynning 2017.

24 Brands 2014, p. 1.

25 Adamsky 2018.

26 Ringsmose and Rynning 2019, pp. 28–29.

27 Haftendorn 1999.

28 NATO 2016, para 11.

29 NATO 2019, para 7.

30 Background interviews, January 2020.

31 Judson 2019. Defender Europe 20 has since been put on hold on account of COVID-19.

32 The budget for EDI is not drawn from the Department of Defense’s base budget, but its Overseas Contingency Operations fund. As the name indicates, this funding stream is contingent but has achieved a remarkable degree of permanence and an equally remarkable size of almost $175 billion per fiscal year (Department of Defense 2019).

33 NATO 2016; Kamp 2019.

34 Binnendijk and Gompert 2019.

35 Ringsmose and Rynning 2019.

36 Rühle and Roberts 2019.

37 Adamsky 2018.

38 Peach 2019.

39 Background interviews NATO headquarters, May and December 2019.

40 A so-called Bi-SC (Strategic Command) Strategic Considerations Report.

41 Background interviews at NATO headquarters, May and December 2019.

42 Gallagher 2019.

43 Gordon and Shapiro 2020.
